# Triboelectric Nanogenerators Based on 2D Materials: From Materials and Devices to Applications

**DOI:** 10.3390/mi14051043

**Published:** 2023-05-12

**Authors:** Yukai Zhou, Jia-Han Zhang, Songlin Li, Hao Qiu, Yi Shi, Lijia Pan

**Affiliations:** Collaborative Innovation Center of Advanced Microstructures, School of Electronic Science and Engineering, Nanjing University, Nanjing 210093, China; dg21230047@smail.nju.edu.cn (Y.Z.); jhzhang@smail.nju.edu.cn (J.-H.Z.); sli@nju.edu.cn (S.L.); haoqiu@nju.edu.cn (H.Q.)

**Keywords:** triboelectric nanogenerators, 2D materials, sensing, mechanical energy harvesting

## Abstract

Recently, there has been an increasing consumption of fossil fuels such as oil and natural gas in both industrial production and daily life. This high demand for non-renewable energy sources has prompted researchers to investigate sustainable and renewable energy alternatives. The development and production of nanogenerators provide a promising solution to address the energy crisis. Triboelectric nanogenerators, in particular, have attracted significant attention due to their portability, stability, high energy conversion efficiency, and compatibility with a wide range of materials. Triboelectric nanogenerators (TENGs) have many potential applications in various fields, such as artificial intelligence (AI) and the Internet of Things (IoT). Additionally, by virtue of their remarkable physical and chemical properties, two-dimensional (2D) materials, such as graphene, transition metal dichalcogenides (TMDs), hexagonal boron nitride (h-BN), MXenes, and layered double hydroxides (LDHs), have played a crucial role in the advancement of TENGs. This review summarizes recent research progress on TENGs based on 2D materials, from materials to their practical applications, and provides suggestions and prospects for future research.

## 1. Introduction

The growing use of fossil fuels such as oil, natural gas, and other non-renewable energy sources in both industrial and daily activities has raised significant concerns regarding the energy crisis [1,2,3,4,5,6]. To mitigate the excessive dependence on these fossil fuels, humans have been exploring and utilizing various sustainable and renewable energy sources, such as solar, wind, nuclear, and tidal power, for several decades. However, there are inherent limitations to the use of these green energy resources. For example, solar energy is constrained by weather, time, and space [7].

In 2006, the first piezoelectric nanogenerator (PENG) was manufactured [8], and later, triboelectric nanogenerators (TENGs) were introduced [9]. These devices utilize the abundant and often overlooked mechanical energy present in daily life, including human activities, mechanical operations, water, and wind. They convert this energy into electrical power for use as an energy collector or to power sensors. In particular, in the era of the Internet of Things (IoT) and with the growing diversification and miniaturization of electronic devices, piezoelectric and triboelectric nanogenerators are becoming more widely used [10]. Compared to PENGs, TENGs utilize two materials with different charged properties to generate significantly enhanced electrical signals. Due to the advantages of a wide range of materials, low cost, industrial scalability, and high energy conversion efficiency, TENGs have considerable application potential in wearable systems, flexible electronics, and artificial intelligence.

TENGs are based on the triboelectric principle. However, due to the negative effects of triboelectricity in industrial production and daily life, such as the accumulation of friction charges on industrial equipment that can detonate surrounding flammable gases [11] and the breakdown of capacitors in high-friction voltage circuits [12], this ancient energy source, which dates back more than 2600 years to ancient Greek civilization, has not been systematically utilized. It was not until the successful design of TENGs that researchers began to pay much attention to these techniques. Although the triboelectric phenomenon is very common in daily life, the physical mechanism behind this universal phenomenon was not thoroughly understood until Wang et al. observed the generation of triboelectric potential by utilizing Kelvin probe force microscopy [13]; they found that electron transfer is the main mechanism of triboelectrification between solids, liquids, and gases. Electron transfer occurs only when the interatomic distance between two materials is forced to be shorter than the normal bond length by rubbing one material against the other through external force. The reduction in the interatomic barrier leads to the overlap of the strong electron cloud between the two atoms in the repulsion region, resulting in electron transition.

TENGs operate in a variety of modes, including contact–separation mode [14], lateral sliding mode [15], single-electrode mode [16], and freestanding triboelectric layer mode [17]. They have advantages in terms of energy collection range, energy conversion efficiency, preparation technology, and device service life. In pursuit of higher output performance, researchers designed different structures to expand the friction area and energy harvesting methods [18]. Proposed device structure designs include arch-shaped [19], disc [20], zigzag [21], and fiber-based 3D friction generators (FB-TENGs) [22] or the design of micropatterns onto the tribo-surfaces [23]. Increasing the charge density of the contact layer [24], introducing more charge–gain groups, or increasing the charge-trapping layer to block the charge combination [25] are also used to increase the output performance of TENGs.

As the key to output performance, the choice of triboelectric material is very important. A range of materials in the triboelectric series have been proposed, including organic polymers, metals, inorganic materials, etc. [26]. Because polymer-based TENGs have an electron with an inclination toward recombination with positive charges induced on the electrode and metal-based TENGs are subject to the issue of the corrosion, it is necessary to use new materials [27]. The fabrication and application of 2D materials provide a promising direction for improvement in material selection for TENGs. Since the successful stripping of two-dimensional graphene, 2D materials with an atomic layer thickness have attracted the attention of researchers around the world. Owing to their excellent electrical properties, transparency, flexibility, and high surface area, 2D materials have great application potential in TENGs [10,26,28], even in a stacked structure.

**Figure 1:**
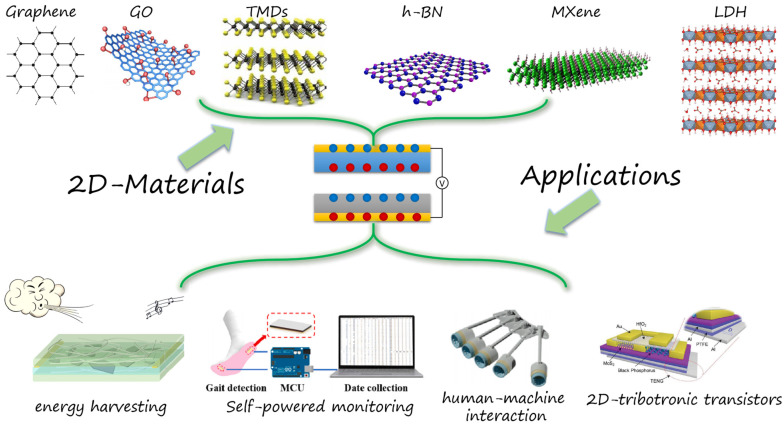
Various 2D materials used in TENGs and practical applications. Reproduced with permission from ref. [29]. Copyright © 2018 Elsevier (MXene); Reproduced with permission from ref. [30]. Copyright © 2020 Wiley-VCH (LDH); Reproduced with permission from ref. [31]. Copyright © 2021 Elsevier (energy harvesting); Reproduced with permission from ref. [32]. Copyright © 2022 Elsevier (self-powered monitoring); Reproduced with permission from ref. [33]. Copyright © 2023 Elsevier (human-machine interaction); Reproduced with permission from ref. [34]. Copyright © 2018 Wiley-VCH (2D-tribotronic transistors).

This review summarizes the exploration and development of 2D materials in the field of TENGs, including the advantages and output properties of different kinds of 2D materials in TENGs. Subsequently, the application of TENGs based on 2D materials in energy harvesting, self-powered monitoring, tribotronic transistors, and other scenarios is also outlined, as shown in Figure 1. Finally, the challenges associated with TENGs based on 2D materials are discussed, and future research directions are proposed.

## 2. 2D Materials Used in TENGs

Since their discovery, 2D materials have been of great interest to researchers. Researchers have attempted to use mechanical stripping, chemical vapor deposition (CVD), the hydrothermal method, etc., to prepare large areas of materials [35]. The variety of 2D materials is also expanding and can be divided into the graphene series, 2D chalcogen compounds, transition metal dichalcogenides (TMDs), metal phosphorous trichalcogenides, metal monochalcogenides, transition metal trichalcogenides, etc. [36,37,38,39]. They are widely used in TENGs, given their respective advantages.

### 2.1. Graphene and Derivatives of Graphene

Graphene, known as the pioneer of 2D material, is one of the most widely studied materials. Graphene has an sp^2^ configuration; it is thin, strong, and flexible, with an ultrahigh electron mobility of 200,000 cm^2^ V^−1^ s^−1^, excellent optical transparency of 97%, and good biocompatibility [40,41,42,43]. Through the study of its electrostatic behavior, it was found that graphene can store a large amount of charge and hold it for a certain time [44,45]. These studies lay the foundation for the application of graphene in TENGs as a friction layer, conductive electrode, or filler.

**Figure 2:**
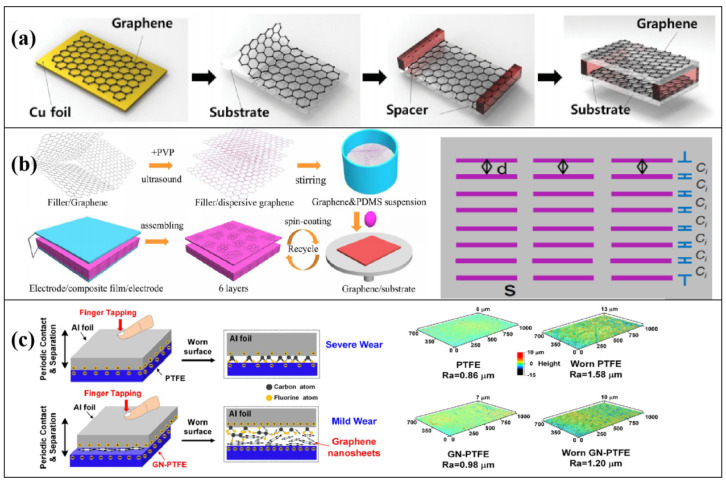
The role of graphene in TENGs: (**a**) first used as a friction layer and electrode in TENGs. Reproduced with permission from ref. [46]. Copyright © 2014 Wiley-VCH; (**b**) Doping to increase capacitance of TENG and reduce dielectric loss. Reproduced with permission from ref. [47]. Copyright © 2017 Elsevier; (**c**) Surface lubrication to prolong service life. Reproduced with permission from ref. [48]. Copyright © 2022 American Chemical Society.

Kim et al. applied graphene to TENGs for the first time in 2014 and fabricated graphene-based TNGs (GTNGs) [46]. A single layer of graphene was grown on Cu foil by chemical vapor deposition and combined with a PET substrate to form a GTNG (Figure 2a). Graphene acts as friction layer and electrode. Then, they designed and fabricated flexible transparent GTNGs by utilizing monolayer (1L), bilayer (2L), trilayer (3L), and quad-layer (4L) graphene utilizing a layer-by-layer transfer technique of 1L graphene grown on Cu foils. For 1L-GTNG, the output voltage and output current density can be achieved at 5 V and 0.5 µA cm^−2^, respectively. The output voltage and output current of randomly stacked 1L, 2L, 3L, and 4L GTNGs decrease as the number of graphene layers increases because as the number of graphene layers increases, the friction caused by folds decreases, and the weak interaction between the randomly stacked graphene layers causes them to easily adhere to the opposite PET surface, resulting in decreased output performance. Additionally, regularly stacked (such as AA/AB, ABC, and ABA) multilayer graphene grown on Ni foils by a CVD method was utilized for the fabrication of GTNGs. Output voltage and output current density increase significantly to 9 V and 1.2 µA cm^−2^, respectively, because the graphene function increases with the number of layers, the charge generated by friction with PET increases, and due to the strong electron relationship between the regularly stacked graphene layers.

In addition to serving as the friction layer and electrode, graphene can also be combined with polymers and metals to modify the material as a filler, improving the output performance and service life of TENGs. Xia et al. successfully embedded aligned graphene sheets (AGS) into PDMS by utilizing the repeated spin-coating (SC) technique to fabricate a TENG based on AGS@PDMS (Figure 2b) [47]. The 2D graphene sheets were arranged in parallel to the surface of the PDMS film due to large shear forces during the SC process. The output current of the TENG based on AGS@PDMS film is three times as much as that of a TENG based on pure PDMS film, and the output power density reaches 4.8 W m^−2^ at a load resistance of 15 MΩ. Compared with a TENG based on PDMS composite films filled with random graphene sheets (RGS), which was made by utilizing the traditional drip-coating (DC) technique, the output performance of AGS@PDMS was also improved. It is assumed that graphene sheets form plane-parallel microcapacitors in series in PDMS. These microcapacitors promote the whole capacitance of the device and enhance the surface tribocharges on PDMS. Recently, Yang et al. found that graphene doping can also lubricate tribomaterials, thereby improving the service life [48]. They embedded graphene nanosheets into polytetrafluoroethylene (PTFE) films. The homogeneous dispersion of graphene nanosheets into the PTFE matrix facilitated the formation of graphene-rich transfer films at the contact interface (Figure 2c). The generation of transfer films could lead to suppression of wear on the worn-out surface.

**Figure 3:**
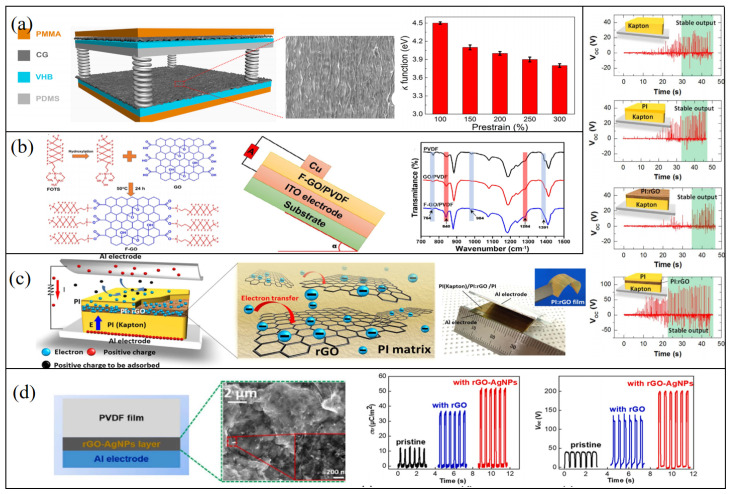
(**a**) Crumpled graphene (CG) acts as an electrode and friction layer, and the folding degree regulates the work function of CG. Reproduced with permission from ref. [51]. Copyright © 2019 Elsevier. (**b**) Functionalized GO was added to improve the dielectric properties of PVDF. Reproduced with permission from ref. [53]. Copyright © 2022 Wiley-VCH. (**c**) Reduced graphene oxide acts as a charge-trapping layer that can quickly establish a stable high output signal. Reproduced with permission from ref. [54]. Copyright © 2017 Elsevier. (**d**) AgNPs can disperse reduced graphene oxide more evenly and enhance the polarization of PVDF. Reproduced with permission from ref. [55]. Copyright © 2020 Elsevier.

As a graphene derivative, crumpled graphene (CG) has high surface roughness and an effective contact area, which is conducive to improving the output performance of TENGs. Based on the same structure, Chen et al. made a flexible TENG out of crumpled graphene [49]. CG and silicone act as friction layers, and CG and planar graphene (PG) act as the electrode layer. CG is a pleated nanostructure formed by transferring PG onto a tape with prestrain. With the increase in prestrain, the degree of folding increases, and the increase in charge density leads to an increase in output voltage and output current density under the same friction. A 1.5 cm^2^ TENG has a 9.3 V output voltage. Furthermore, TENGs an operate in hybrid compressive and stretching modes, revealing their tolerance to practical tensile and twist motions. It has also been reported that the folding degree can control the output performance by controlling the work function of CG [50]. Researchers verified this theoretical calculation by analyzing the UPS spectra of CG with different folds (Figure 3a) [51]. The designed friction layer for the TENG was CG and PDMS with microstructures, and the electrode was CG. Following adjustment of the degree of folding, the output voltage, current, and power density of the TENG were 83 V, 25.78 μA, and 0.25 mW cm^−2^, respectively, with 300% prestrain. Graphene oxide (GO) consists of a carbon hexagonal ring of both aromatic (sp^2^) and aliphatic (sp^3^) orbital hybrids. Although GO has low electrical conductivity compared to pristine graphene, the presence of oxygen-containing functional groups makes it easier to functionalize GO. It has great potential to improve the output performance of TNEGs [52]. Vu et al. utilized the blade-coating process to fabricate a liquid–solid TENG based on functionalized graphene oxide (F–GO) and polyvinylidene fluoride (PVDF) composite membranes containing F–GO [53]. The surface of GO nanosheets was functionalized through covalent grafting utilizing FOTS. The significant specific interaction between oxygen-containing groups on the GO and fluorine in PVDF increased the polar phase of the membrane (*β*-phase) (Figure 3b). Thus, the dielectric properties of the PVDF were improved, and the transferred charge density of the membrane increased, resulting in the improved electrical output of the TENG.

It has been reported that due to the unique electronic and morphologic properties of reduced graphene oxide (rGO), it can efficiently capture external electrons with nonvolatile electron-trapping properties [56,57]. Therefore, researchers improved the output performance of a TENG by introducing rGO as an electron trap into the friction layer to inhibit tribological electron loss. Wu et al. introduced rGO as an electron trap in the common friction layer of a TENG for the first time [54]. They embedded rGO in the negative friction layer polyimide (PI). The negative friction layer is a PI (Kapton)/PI: rGO/PI stacked structure (Figure 3c). They used fluorescence methods and absorption spectroscopy to explore the mechanism of the action of rGO. The enhanced excitation of PI:rGO nanocomposites shows that rGO sheets can facilitate the charge transfer process, whereby electrons are transferred from electron-donor diamine to electron-receiving rGO sheets (charge transfer), then captured in the rGO sheet (charge capture). The red shift of absorption spectra indicates that rGO can reduce the energy gap of CT transition in PI, which can originate from electron transfer from the electron-donating diamine to the electron-accepting rGO sheet. The addition of an electronic trap enhances the output performance of TENGs significantly; the maximum output voltage was 190 V for an rGO with 6.5 μm/16.8 wt%. The maximum output power density reached 6.3 Wm^−2^. Due to the effective inhibition of the recombination of negative charge and positive charge, stable voltage output can be quickly established for TENGs with a PI:rGO layer (Figure 3c). Additionally, the electron density under low operation frequencies is almost the same as that under high operation frequencies. Furthermore, Jiang et al. found that the introduction of AgNPs onto the rGO layer can enhance the polarization of the tribomaterial through the strengthening of the interfacial polarizations between the AgNPs and graphene (Figure 3d) [55]. The successful introduction of electronic traps provides a more feasible way to improve the performance of TENGs.

In conclusion, graphene is widely used in the electrodes and friction layers of TENGs due to its extremely high electron mobility, flexibility, and transparency, and its derivatives also play an important role in regulating the output properties of TENGs. In addition to being a 2D film, graphene has the advantages of good electronic/mechanical properties and can be subjectively synthesized into other macroscopic forms (e.g., zero-dimensional (0D) graphene quantum dots, 1D graphene fibers, and 3D graphene foam) [58,59,60]. It also has great potential to improve the output performance of TENGs. In-depth research on graphene also lays a foundation for the application of other 2D materials in TENGs.

### 2.2. Hexagonal Boron Nitride (h-BN)

**Figure 4:**
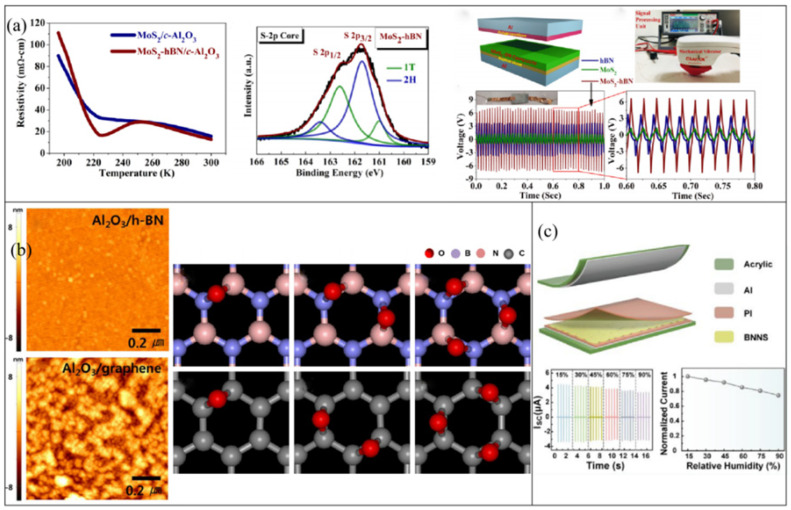
(**a**) H-BN interacts with MoS_2_ to create a stable biphase, enhance charge capture in TENGs, and regulate the work function. Reproduced with permission from ref. [67]. Copyright © 2019 American Physical Society. (**b**) High-quality Al_2_O_3_ film successfully grows on the BN/graphene interface and acts as a friction layer for the TENG. Reproduced with permission from ref. [68]. Copyright © 2015 Elsevier. (**c**) BNNSs enhance the TENG output as a charge-trapping layer. Reproduced with permission from ref. [70]. Copyright © 2022 American Chemical Society.

Hexagonal boron nitride, a graphene-like 2D material, has the same hexagonal honeycomb structure as graphene [61]. B atoms and N atoms are connected by strong covalent bonds, which causes some B–N bonds to have ionic bond properties after being ionized, resulting in a wide band gap of up to 6 eV, meaning that the material not only has the characteristics of high thermal conductivity, high insulation, and structural stability [62] but can also be used for the hybridization of other 2D materials to prepare high-performance composite materials [63,64]. In addition, h-BN can be modified by doping, surface functionalization, defect engineering, and other methods [65,66]. Parmar et al. used the interaction between h-BN and MoS_2_ to fabricate hybrid structures and prepared a MoS_2_-hBN composite film by pulsed-laser deposition [67]. Due to the specific pulsed-laser deposition (PLD) growth dynamics and strong interlayer coupling between MoS_2_ and hBN, there is a stable biphase in MoS_2_, 1T (conducting), and 2H (semiconducting). The resistance shows a semiconductor–metal–insulator transition with temperature that is not present in MoS_2_ films (Figure 4a). Researchers inferred that interfaces of h-BN and related materials seem to create locations for trapped charges due to the formation of interfaces with varying work functions, which can alter the contact potential difference (CPD) and impact electron transfer processes. A TENG utilizing MoS_2_-h-BN composite film exhibits peak-to-peak output voltage that is more than two times and six times greater than that of pure MoS_2_ and h-BN materials, respectively.

H-BN can be used as a buffer layer to grow high-quality, high-k dielectric films on graphene due to its similar crystal structure and similar lattice constants to those of graphene. Han et al. successfully grew high-quality Al_2_O_3_ thin films on h-BN/graphene structures [68]. H-BN grown on graphene utilizing CVD is atomically flat and free of dangling bonds and charge traps with sp^2^ bonding configurations. In particular, there are similar ionic bonding properties between the Al^+^/O^−^ bond and the B^+^/N^−^ bond (Figure 4b). These factors lead to the preparation of high-quality Al_2_O_3_ films. Using h-BN nanosheet as passivation layer can also greatly reduce the loss of carrier mobility caused by graphene-based film preparation. A TENG with graphene-Al_2_O_3_/BN/graphene exhibits higher electrical properties than one without BN.

Similar to other 2D materials, h-BN can be hybridized with polymers to improve their dielectric properties and electron-trapping capabilities. Bhavya et al. prepared 2D hexagonal boron nitride nanosheets (BNNSs) by utilizing ultrasound-assisted liquid-phase exfoliation [69]. It was coated on biaxially oriented polyethylene terephthalate (BoPET) as a negative friction layer, with paper as the positive layer for the fabricated TENG. The addition of BNNSs can significantly increase the dielectric constant of the BoPET substrate, improve the charge capture ability of the system, and increase the surface charge density, resulting in better electrical output performance of TENGs. It exhibits an impressive power output that is 70 times higher than that of simple a BoPET–paper TENG assembly. Utilizing the same preparation method and gain mechanism, Pang et al. embedded BNNS in a PI film and fabricated a TENG based on a sandwich-structured PI/BNNS/PI nanocomposite film (named PBP) (Figure 4c) [70]. The power density of the composite film was 15 times higher than that of that of a TENG without a BNNS interlayer (PP-TENG). Furthermore, with the increase in charge retention ability, the PBP-TENG also maintained good output performance in a relatively high-humidity environment.

### 2.3. Transition Metal Dichalcogenides (TMDs)

TMDs are two-dimensional semiconductors that can be represented by MX_2_, where M is the transition metal atom and X represents the chalcogen atoms (S, Se, Te, etc.). Compared to the use of graphene as a gapless semiconductor, the band gap of TMDs is controlled by their thickness, except that the 2D materials have the same advantages of flexibility, transparency, and stability. Furthermore, the direct/indirect transition also varies with thickness [71,72]. Owing to these characteristics, TMDs are widely used in energy harvesting, integrated circuits, flexible electronics, and other fields.

**Figure 5:**
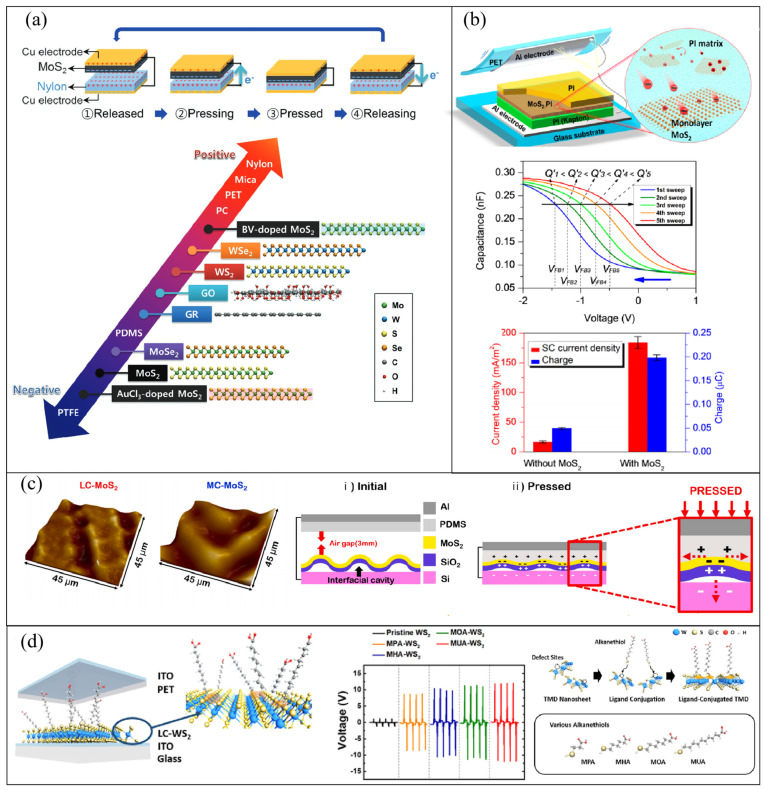
(**a**) Triboelectric series including 2D materials. Reproduced with permission from ref. [73]. Copyright © 2018 Wiley-VCH. (**b**) MoS_2_ acts as a charge-trapping layer to enhance the output performance of TENGs. Reproduced with permission from ref. [76]. Copyright © 2017 American Chemical Society. (**c**) The use of MC-MoS_2_ as the friction layer increases the contact area. Reproduced with permission from ref. [79]. Copyright © 2020 Elsevier. (**d**) The surface defects of friction layer WS_2_ were modified by thiol groups to improve the output of LC-WS_2_ TENGs. Reproduced with permission from ref. [81]. Copyright © 2021 American Chemical Society.

For this kind of new material in the triboelectric series, Seol et al., utilizing the concept of a triboelectric nanogenerator, conducted the first systematic study of their triboelectric charging behavior, including graphene, GO, WS_2_, MoSe_2_, WSe_2_, and MoS_2_ [73]. They used nylon and MoS_2_ as friction layers and observed the polarity of the voltage. They found that MoS_2_ was negatively electrified when contacted with nylon. Similarly, they prepared several TENG combinations between MoS_2_ and six different well-known materials in the triboelectric series: (−) polytetrafluoroethylene (PTFE), polydimethylsiloxane (PDMS), polycarbonate (PC), polyethylene terephthalate (PET), mica, and nylon (+). The MoS_2_–PTFE pair exhibited the opposite polarity to that of the MoS_2_–nylon pair, and all other pairs were identical to nylon. Therefore, MoS_2_ lies between PTFE and PDMS in the triboelectric series. Based on the same method, the authors tested other materials and defined the relative positions of 2D materials in the conventional triboelectric series (Figure 5a). All the 2D materials used in the study are located near the negative side of the triboelectric series. It is worth noting that in order to avoid the influence of other factors on the output, all 2D materials used in the study had the same thickness and surface roughness and were prepared by chemical exfoliation of bulk flakes in a liquid phase. To further verify the results, the authors calculated the effective work function utilizing Kelvin probe force microscopy (KPFM) and via first principles simulations. They also found that the triboelectric polarity of MoS_2_ did not vary with the thickness, preparation method, or polytype. On the contrary, its position in the friction series can be successfully regulated by chemical doping.

Similar to rGO, TMDs materials, represented by MoS_2_, also have the ability to capture charge and inhibit the recombination of electron and positive charge due to drift and diffusion [74,75]. Wu et al. studied the effect of embedding MOS_2_ in the friction layer as the triboelectric electron-acceptor layer on enhancing the output performance of TENGs [76]. Under the action of ultrasound, they used liquid-phase peeling conduction to peel off the bulk MoS_2_ in an organic solvent to obtain a monolayer MoS_2_. The electrodes were an Al electrode and a negative friction layer with a PI (Kapton)/MoS_2_:PI/PI stacked structure (Figure 5b). The TENG with monolayer MoS_2_ as an electron-acceptor layer had a power density of up to 25.7 W m^−2^, which is 120 times larger than that of the device without monolayer MoS_2_. The authors also fabricated a floating-gate metal-insulator semiconductor device (MIS) with MoS_2_ to prove the ability to capture charge. In contrast to rGO, band the gap of monolayer MoS_2_ is as large as 1.8 eV, and the interface trap states are distributed in the band gap. Therefore, as the number of voltage sweeps increases, the number of electrons captured in the interface trap states of the MoS_2_ gradually increases, leading to a shift in the C–V curves (Figure 5b).

For the friction layer of TENGs, the surface microstructure and proper friction degree are of great help to improve the performance. However, the traditional preparation methods, such as mechanical stripping and CVD, are subject to limitations with respect to the controllability of surface morphology [77,78]. To solve this problem, Park et al. used pulsed-laser-directed thermolysis to prepare a surface-crumpled MoS_2_ (Figure 5c) [79]. This technique involves the application of internal stress to MoS_2_ crystal by adjusting its morphological structure. The separation of the underlying SiO_2_ thin film layer from the Si substrate causes interfacial cavities, resulting in wrinkles. With the increase in laser irradiating fluence, the flat surface of MoS_2_ (F-MoS_2_) gradually develops fewer wrinkles (LC-MoS_2_); furthermore, the small wrinkles merge with one another, cauterizing the most crumpled structure (MC-MoS_2_). The MC-MoS_2_ TENG device generates ~40% more power than a flat F-MoS_2_ device. The maximum open-circuit voltage and short-circuit current were 25 V and 1.2 μA, respectively. The authors believed that the existence of wrinkles increases the surface roughness and friction area (Figure 5c) and that the application of tensile strain to MoS_2_ causes the distance between atoms to increase, as well as lattice expansion. Therefore, the location of valence bandwidth and Fermi level decreases, resulting in an increase in the work function of MoS_2_. These factors combine to improve the performance of surface-crumpled MoS_2_ TENGs.

Surface defects easily occur in the process of TMD preparation. For example, WS_2_ produces sulfur vacancies on the basal planes or edges, resulting in a decrease in carrier mobility and charge density [80]. This greatly affects the performance of the TENGs based on TMDs. Recently, Kim et al. designed the surface of TMDs utilizing thiolated ligands (LC-WS_2_) of varying alkane chain lengths and used them in conjugation in order to create TMD based TENG devices with improved output performance (Figure 5d) [81]. Utilizing the high affinity between the defect sites and thiol groups, the thiol-containing ligands, including mercaptopropionic acid (MPA), mercaptohexanoic acid (MHA), mercapto-octanoic acid (MOA), and mercaptoundecanoic acid (MUA), were used as functionalizing materials to modify the surface of WS_2_ nanosheets containing defects. ATR-IR and XPS were used to analyze WS_2_ surface conjugates with various kinds of thiol-containing ligands. The output voltage and power density of the LC-WS_2_ TENGs were respectively 12.2 V and 138 mW m^−2^, respectively. The authors attributed the improved performance to the increase in surface charge density on the one hand and the p-type doping effect induced by thiolated ligand conjugation on the other hand, causing the LC-WS_2_ to exhibit a higher work function. The stability of WS_2_ in air was also enhanced.

In general, in addition to the surface modification and electron capture capabilities of graphene and its derivatives, TMDs can also be used for the preparation of various detectors, including for detection of light, humidity, biological signals, etc., owing to their band-gap-adjustable properties, and can be combined with TENGs to broaden their application range.

### 2.4. MXenes

The precursors of MXenes are transition metal carbides or nitrides. The general formula is M_*n*+1_AX_*n*_, where *n* = 1, 2, 3; M is the excessive metal element; A represents the III or IV group 2 element; and X represents carbon or nitrogen. After selective etching of layer A, the M–A bond is replaced to obtain MXene, the general formula of which is M_*n*+1_X*_n_*T*_x_*, where T_x_ is the surface termination. As a new two-dimensional layered material, MXene has excellent physical and chemical properties, including high mechanical strength, good electrical conductivity (more than 20,000 S cm^−1^), abundant surface functional groups, and high negative zeta potential [82,83]. It has great potential for use in energy devices [84].

**Figure 6:**
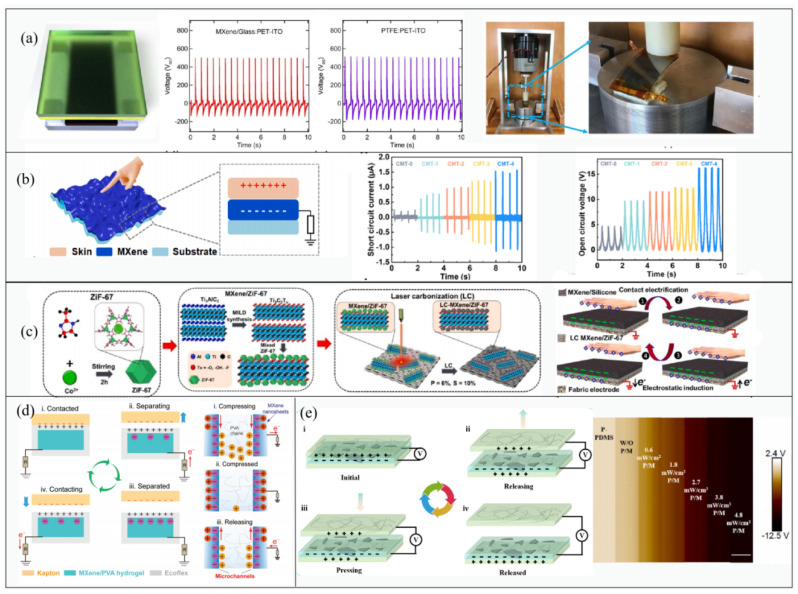
(**a**) Ti_3_C_2_T_x_ (MXene) was first used as a friction layer in a TENG. Reproduced with permission from ref. [29]. Copyright © 2018 Elsevier. (**b**) Wrinkled MXene was used to increase the contact area of the friction layer. Reproduced with permission from ref. [85]. Copyright © 2022 Elsevier. (**c**) A non-contact TENG with LC-MXene/ZiF-67 as the electron capture layer. 1–4 represent the electricity generation of TENG. Reproduced with permission from ref. [86]. Copyright © 2022 Elsevier. (**d**) The SVP model (i–iii on right of picture) generated between MXene and hydrogel enhances the TENG output. i–iv on the left of picture represent the electricity generation of TENG. Reproduced with permission from ref. [87]. Copyright © 2021 Wiley-VCH. (**e**) Light enhanced the triboelectric properties of MXene. i–iv represent the electricity generation of TENG. Reproduced with permission from ref. [31]. Copyright © 2021 Elsevier.

Dong first attempted to use MXene materials for the negative triboelectrification layer and electrode of a TENG. Ti_3_C_2_T_x_ MXene has a highly electronegative surface due to halogen group (−F) and oxygen-containing terminating functional groups (Figure 6a) [29]. Compared with the output voltage of a TENG, a single-electrode mode with PTFE as the friction layer, Ti_3_C_2_T_x_ has a similar output, proving that Ti_3_C_2_T_x_ MXene is comparable to PTFE in the triboelectric series. An alternative was found for electronegative polymers (PTFE, PDMS, and FEP) that are not conductive and limited to single-electrode mode operation. A MXene-PET TENG (2.5 × 5 cm^2^) generated potential differences as high as ~650 V with a maximum peak power of ~0.65 mW. The MXene-PET TENG was tested by clamping at an angle of 30° and generated an output of approximately 40 V when a force of approximately 1 N was applied (Figure 6a). This TENG can be used to collect energy when the fingers are bent. Utilizing the same materials, Cao et al. dripped Ti_3_C_2_T_x_ ink onto an expanded latex balloon; after the ink dried, the balloon was slowly shrunk to produce a wrinkled MXene film. Then, a single-electrode-mode stretchable TENG was fabricated (Figure 6b) [85]. Owing to its crumpled structure, the maximum area strain and linear tensile ratio of the MXene TENG were 2150% and 400%, respectively. The authors found that both the increased MXene content in the ink and the fold structure had a positive effect on the output, but the latter dominated. Based on the stretchable TENG, they fabricated a highly sensitive pressure sensor.

Salauddin et al. used lasers to carbonize MXene composites to create an LC-MXene/ZiF-67 nanocomposite layer and used it as an intermediate layer to conserved electrons to prepare a non-contact mode (CNM) TENG for the first time (Figure 6c) [86]. The LC-MXene/ZiF-67 nanocomposite layer possesses a porous structure with a high charge density, which helps to maintain surface charges for extended periods of time, in addition to increasing the surface potential. The ZiF-67 results in a unique porous structural framework of the nanocomposite, as well as excellent chemical stability and consistent porosity. Carbonization significantly improves the electron capture ability of the nanocomposite, which helps to maintain the surface charge for a long time, while improving the surface potential. The authors used MXene/silicone nanocomposite as charge-generating layer (CGL) and constructed microstructures on its surface to increase the effective contact area. They also enhanced the output power density utilizing fabric surface textures. In a CNM-TENG, the surface of the MXene/silicon nanocomposite was negatively charged before the human hand touched the finger. Due to the distance between the intermediate layer with CGL and the human hand, electrical impulses were generated during its motion. As the hand moved, electric signals were produced as a result of this relative distance. The authors also investigated the effects of laser power, laser speed, ratios of LC-MXene/ZiF-67, and distance on the performance of the TENG.

Luo et al., fabricated a high-performance excellent self-healing and stretchable TENG by mixing Ti_3_C_2_T_x_ MXene nanosheets into the hydrogel (Figure 6d) [87]. The incorporation of nanosheets resulted in the formation of microchannels on the surface in contact with hydrogels, which can not only improve the conductivity of hydrogels by improving ion transport and, as a cross-linking agent, improve the mechanical properties of composites but also enhances the triboelectric effect through a streaming vibration potential mechanism. The authors encapsulated the MXene/PVA hydrogel with Ecoflex silicone rubber and used the hydrogel as an electrode to form a TENG in single-motor mode with Kapton. When pressure was applied to the MH-TENG, in addition to the potential difference generated by the contact between friction layers of Ecoflex and Kapton, the streaming vibration potential model formed by the interface between MXene and the hydrogel also generated additional potential difference as the material was compressed, which increased with the degree of stretching (Figure 6d). Doping of MXene nanosheets provided the TENG with good mechanical stability and stretchability, in addition to producing greater output performance. This TENG can be used as a handwriting recognition system through the electrical signals generated by compressing and contacting.

It is worth noting that in addition to serving as an excellent electronegative conductor, MXene is also a photosensitive material [88]. MXenes are made up of disordered layers of crystals that are several hundred nanometers in size. They have highly concentrated edges and small gaps that can effectively relieve plasmonic momentum restrictions, thereby facilitating the generation of energized hot electrons [89,90]. Based on this property, Liu et al. created a light-enhanced stretchable TENG (Figure 6e) [31]. These one-structure-layer PDMS/MXenes-based TENGs can simultaneously harvest mechanical and light energy. The conductive electrode and friction layer were prepared by mixing Ag nanowires and MXene nanosheets into PDMS elastomer, respectively. Under light exposure, the device showed significant improvements in open-circuit voltage (145 to 453 V) and short-circuit current (27 to 131 μA), resulting in an instantaneous light power conversion efficiency of 19.6%. The production of energized hot electrons can greatly enhance the surface charge density and dielectric constant of PDMS/MXene composite films. As the light irradiation power increased, the surface potential of the films also increased.

### 2.5. Layered Double Hydroxides (LDHs)

An LDH is a layered compound with a hydrotalcite structure. It consists of several layers of positively charged ions and anions with balanced charges between the layers. Its molecular formula can be expressed as [M^2+^_1−*x*_M^3+^*_x_*(OH)_2_]^x+^[A*_x/n_*^n−^]^x−^·mH_2_O, where M^2+^ is the divalent metal oxygen ion (Mg^2+^,Ga^2+^,Ni^2+^), M^3+^ is a substituted trivalent metal cation (Al^3+^,Fe^3+^,Cr^3+^), and A^n−^ is the interlayer anion (OH^−^,Cl^−^,CO_3_^2−^,SO_4_^2−^) [91]. LDHs exhibit cationic tunability, anion exchange capability, and biocompatibility, rendering them versatile materials for drug delivery, energy storage, catalysis, and luminescence. Moreover, the abundant hydroxyl groups on the surface of LDHs enable facile functionalization or integration with other materials to achieve tailored properties [30,92].

**Figure 7:**
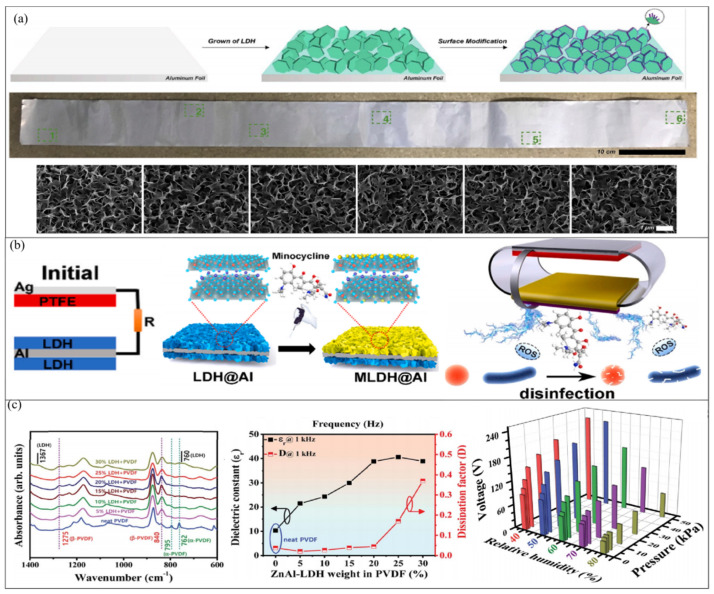
TENGs based on LDHs. (**a**) A high-performance water-driven triboelectric nanogenerator based on modified LDH. SEM images of modified LDHs at six different regions where marked in green squres in the picture above. Reproduced with permission from ref. [93]. Copyright © 2020 Elsevier. (**b**) A TENG patch used to inhibit bacterial growth and promote cell reproduction. Reproduced with permission from ref. [94]. Copyright © 2021 Elsevier. (**c**) Flexible pressure sensor and humidity sensor based on a TENG. Reproduced with permission from ref. [95]. Copyright © 2021 The Royal Society of Chemistry.

Recently, Cui et al. reported the successful in situ growth of meter-scale Mg-Al LDHs on Al electrodes using a bottom-up method [93]. By utilizing PFTS as a functionalizing agent, an LDH was modified to enhance the surface roughness and hydrophobicity and to serve as a friction layer in contact with water droplets (Figure 7a). This enabled the fabrication of a high-performance water-driven triboelectric nanogenerator (WD-TENG) capable of collecting energy generated by water droplets passing over a tilted surface. The WD-TENG exhibited an output voltage of 13 V and a current density of 1.6 μA cm^−2^. Notably, the acidity and alkalinity of water droplets had little influence on the performance output, providing meaningful guidance for practical applications in diverse rainfall environments and locations. Later, building on this preparation method, Du et al. designed a flexible TENG patch with a surface-engineered electrode featuring Mg-Al layered double hydroxide as a smart drug container and friction layer [94]. This patch was utilized to inhibit bacterial growth and promote wound healing through miniaturized electrical stimulation (ES) and drug delivery. The TENG with a surface-engineered LDH@Al electrode (STENG) produced an alternating-current (AC) low-intensity electric field (LIEF) capable of generating ES (Figure 7b). This disrupted the bacterial membrane, improving permeability and causing the loss of inside fluid, resulting in the inhibition and killing of bacteria. Moreover, with prolonged ES action time, the H_2_O_2_ content increased, which also had an inhibitory effect on bacteria. Furthermore, minocycline was incorporated within the LDH@Al, and the AC LIEF accelerated its release, allowing the drug to act on damaged bacteria. Moreover, the AC LIEF from SETENG was found to promote cell proliferation. Together, these effects resulted in the patch effectively inhibiting wound bacteria (~96.7%) and promoting skin tissue repair.

Like other 2D materials, LDHs can also be utilized for material modification. Ippili et al. prepared a composite material using a spin-coating method, which was subsequently applied to the friction layer of a TENG [95]. LDHs can serve as excellent polar materials and can be combined with PVDF polymer (Figure 7c). Through the formation of hydrogen bonds between PVDF and hydroxyl groups (–OH), their interaction facilitates the formation of a self-polarized *β*-PVDF phase. Consequently, the dielectric properties of the composite films are significantly improved. The dielectric constant (*ε*_r_) and dissipation factor (*D*) of the composite films were tested at varying concentrations of LDH, and it was observed that these properties increase with an increase in LDH content. In order to achieve a higher output from TENGs, a high dielectric coefficient and low dissipation factor are required, which leads to an increase in charge storage time and a decrease in leakage current. Therefore, the optimal LDH content was determined to be 20 wt%. The resulting composite exhibited excellent pressure sensitivity, with low- and high-pressure zones of 13.07 V Pa^−1^ and 1.65 V Pa^−1^, respectively. Additionally, due to the low level of charge transfer between the surfaces of the two friction layers at higher humidity, the composite material can also be utilized as a humidity sensor with a response rate of 259.4% in voltage detection mode.

**Figure 8:**
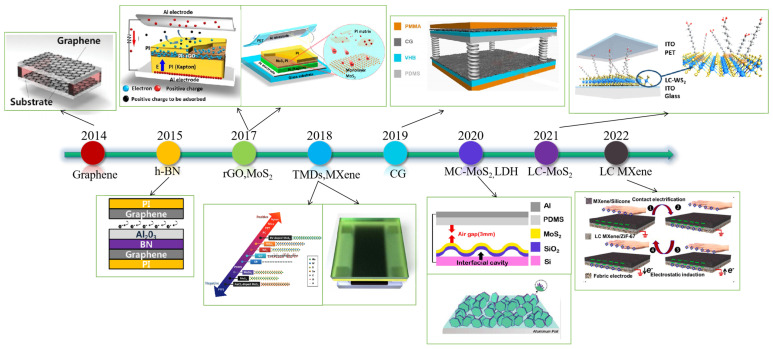
Timeline of major milestones of the TENGs based on 2D materials. Reproduced with permission from ref. [46]. Copyright © 2014 Wiley-VCH; Reproduced with permission from ref. [68]. Copyright © 2015 Elsevier; Reproduced with permission from ref. [54]. Copyright © 2017 Elsevier; Reproduced with permission from ref. [76]. Copyright © 2017 American Chemical Society; Reproduced with permission from ref. [73]. Copyright © 2018 Wiley-VCH; Reproduced with permission from ref. [29]. Copyright © 2018 Elsevier; Reproduced with permission from ref. [51]. Copyright © 2019 Elsevier; Reproduced with permission from ref. [79]. Copyright © 2020 Elsevier; Reproduced with permission from ref. [93]. Copyright © 2020 Elsevier; Reproduced with permission from ref. [81]. Copyright © 2021 American Chemical Society; Reproduced with permission from ref. [86]. Copyright © 2022 Elsevier.

The above summarizes the application of different two-dimensional materials in TENGs in recent years. Such materials can be directly used as a friction layer, electrode, or dopant to compound with other materials; they can also be used as a charge-trapping layer to enhance the output performance. The performance of these TENGs based on 2D materials is summarized in Table 1. Figure 8 shows a summary of the progress of TENGs based on 2D materials over time.

## 3. Applications of TENGs Based on 2D Materials

In the preceding Introduction and discussion, we provided a summary of the use of 2D materials in the context of triboelectric nanogenerators. Specifically, 2D materials have been primarily employed as friction layers and electrodes in TENGs, particularly those exhibiting high electrical conductivity and those positioned at side positions within the triboelectric series, such as MXene and graphene. The exceptional mechanical stability, smooth surface, and facile surface functionalization of 2D materials lead to TENGs that possess enhanced electrical output and longer operational lifetimes in comparison to those utilizing other triboelectric materials. Moreover, due to the atomic layer thickness, the resulting device is lighter and thinner, even if stacking structures are used [7,10,38]. Additionally, extensive investigations have been conducted into the electron-capturing ability of 2D materials, with significant applications arising from the incorporation of these materials into other triboelectric materials. This integration enables regulation of the dielectric properties, work function, and other features of the resulting hybrid materials, ultimately increasing the work function difference and surface charge density and positively impacting the output performance of TENGs. In the following section, we focus on summarizing the practical application of TENGs based on 2D materials.

### 3.1. Energy Harvesting

**Figure 9:**
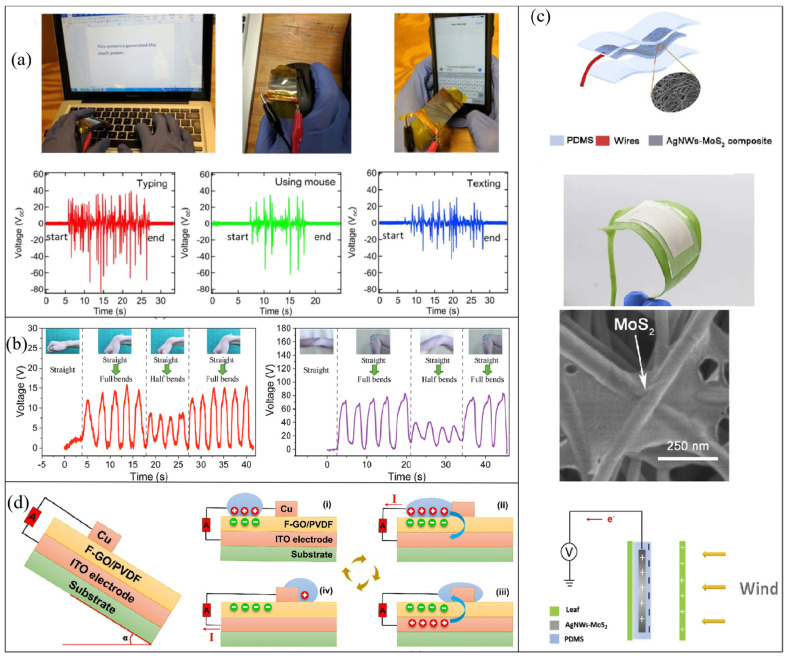
TENGs based on 2D materials used for energy harvesting. (**a**) Collection of the mechanical energy of typing on a keyboard, using a mouse, and composing a text message. Reproduced with permission from ref. [29]. Copyright © 2018 Elsevier. (**b**) Collection of the mechanical energy generated by bending fingers, elbows, and wrists. Reproduced with permission from ref. [87]. Copyright © 2021 Wiley-VCH. (**c**) A flexible TENG attached to a blade to collect wind energy and measure wind speed. Reproduced with permission from ref. [96]. Copyright © 2019 Elsevier. (**d**) Collection of water energy. i–iv represent the electricity generation of TENG and A is the ammeter. Reproduced with permission from ref. [53]. Copyright © 2022 Wiley-VCH.

Energy harvesting was the original design intention of TENGs. With the increasing demand for energy and the proliferation of mobile intelligent devices, these miniature energy harvesting devices, which can maintain high output at low frequencies, have a wide range of potential applications in daily life. Due to the flexibility, robustness, and stability of the materials, TENGs based on 2D materials can be adhered to the surface of the human body or other complex structures to efficiently collect mechanical energy generated by human movements, wind, liquid flow, and other activities, which are used in many of the works described above. For instance, Chen et al. attached a TENG based on crumpled graphene to the finger joint [49]. When the bending angle changed from 30° to 90°, the device produced an output voltage of 32 to 56 mV, and the bending rate could also be measured. Dong et al. first utilized MXene to fabricate a TENG, which they attached to the thumb joint to capture energy surges in the joint, such as editing a text message or tapping a mouse (Figure 9a) [29]. The device showed a significant open-circuit voltage ranging from −80 to + 40 V. Luo et al. designed an MH-TENG that can convert mechanical energy generated by human joint activities into electric energy [87]. Different degrees of extrusion and stretching of the TENG can produce different output voltages when different joints are bent and restored, which can be used to monitor human posture (Figure 9b).

Drawing inspiration from natural energy harvesting, Lan et al. developed a stretchable triboelectric nanogenerator capable of converting wind energy into electrical output while conforming to the surface of a plant leaf (Figure 9c) [96]. The authors utilized a combination of 1D AgNWs and 2D metallic MoS_2_ nanosheets to form highly conductive and flexible composite films as electrodes. The composite film was subsequently covered and packaged with PDMS, with the nanosheets and AgNWs tightly bonded by electrostatic and capillary forces. The inclusion of metallic MoS_2_ nanosheets served to cover the AgNWs network, reducing strain distribution and sliding during large tensile deformation, thereby promoting good electrical output and better attachment to the blade. Additionally, the output voltage signal of the stretchable TENG varies with blade vibration frequency at different wind speeds, making it a valuable tool for harvesting and converting wind energy (Figure 9c).

Water energy harvesting represents a great innovation of TENGs in the field of energy harvesting. It generates electricity using water in direct contact with the friction material in the form of droplets or streams or by collecting the mechanical energy generated by water in motion, such as waves. The former uses the principle of liquid–solid contact electrification, and the latter, with a fully enclosed structure, makes use of mechanical impact and vibration under the action of water waves [97,98]. Ocean energy, also known as blue energy, is an important flied of TENGs in water energy collection. Among various forms of ocean energy, water wave energy stands out as a renewable source that is not affected by seasonal changes and has a significant reserve. It is estimated that the wave energy around coastlines can reach 2–3 TW (1 TW = 10^12^ W) [99]. However, the electromagnetic generators (EMGs) currently used for wave energy harvesting are expensive, bulky, and inefficient, hindering their effective utilization. In contrast, TENGs offer a new energy harvesting method that is lightweight, simple, low-cost, and easy to package and is particularly suitable for collecting low-frequency (<5 Hz) wave energy in complex ocean environments [100,101,102]. By forming a TENG network, water wave energy can be collected on a large scale, effectively alleviating energy problems [97]. In recent years, TENGs based on two-dimensional (2D) materials have been explored for harvesting of water energy. For instance, Vu et al. modified polyvinylidene fluoride (PVDF) by incorporating functionalized graphene oxide (GO) as a friction layer in contact with water droplets [53]. They employed an inclined structure to make water droplets slide on the friction layer and generate electricity through solid–liquid contact electrification (Figure 9d). A single water droplet can produce an output of 0.14 mW, maintaining 90% of the output voltage after more than 4000 cycles. Based on the same principle, Cui et al. also managed to capture the triboelectric energy generated by water droplets sliding along an inclined surface. Layered double hydroxides (LDHs) grown in situ were used as the triboelectric layer, and modified LDHs were superhydrophobic. The peak output voltage was about 13 V, and the current density was 1.6 μA cm^−2^.

### 3.2. Self-Powered Monitoring Systems

TENGs are based on the fascinating principle of triboelectricity. This principle describes a unique electromechanical coupling effect that facilitates not only energy harvesting but also the creation of pressure sensors and arrays. The output signals of TENGs are closely related to the applied pressure and speed, enabling the design of sophisticated wearable sensing systems that can effectively monitor human activities, especially in electronic skin, human–machine interaction, and wisdom medical sectors [103,104]. The integration of TENG technology based on 2D materials in wearable sensing systems eliminates the need for an external power supply, offering improved portability and stability, which presents a significant advantage over other wearable monitoring systems. This feature reduces costs while simultaneously improving the user experience [105,106]. The development of miniaturized self-powered wearable pressure monitoring systems is an exciting area of research, with immense potential for the advancement of the IoT ecosystem. The growing interest in this field has attracted an increasing number of researchers to join in.

**Figure 10:**
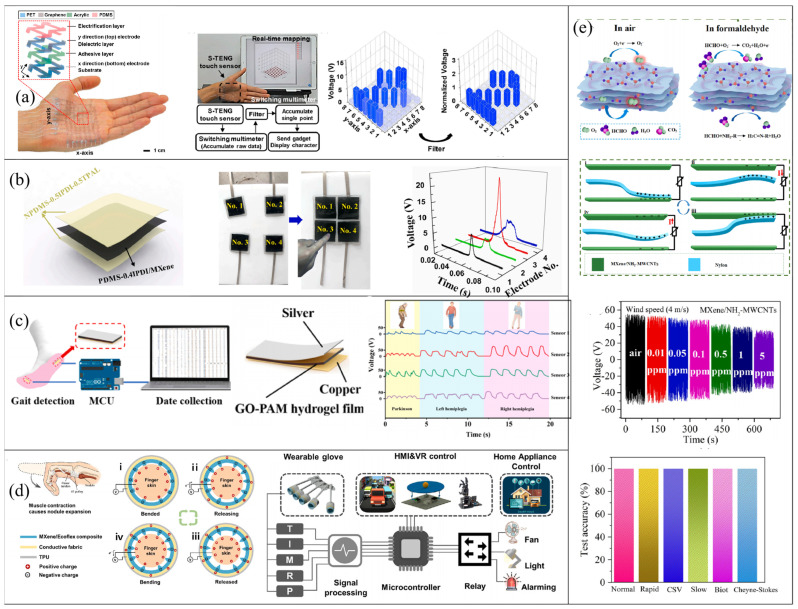
TENGs based on 2D materials used for self-powered monitoring systems. (**a**) A touch sensor can clearly detect the touch of a single point and continuous sliding and display a character using a real-time trajectory mode. Reproduced with permission from ref. [107]. Copyright © 2019 Elsevier. (**b**) A self-powered sensor array can be used for real-time monitoring of finger pressure and position. Reproduced with permission from ref. [108]. Copyright © 2020 Elsevier. (**c**) A sensor array placed on an insole and used to identify and monitor walking posture. Reproduced with permission from ref. [32]. Copyright © 2022 Elsevier. (**d**) 3D-printed self-powered smart gloves for human–computer interaction. i–iv represent the electricity generation of TENG. Reproduced with permission from ref. [33]. Copyright © 2023 Elsevier. (**e**) Sensor used for respiratory behavior monitoring and recognition and formaldehyde gas sensing. Reproduced with permission from ref. [109]. Copyright © 2022 Elsevier.

Tactile monitoring is an important task that electronic skin strives to achieve. Lee et al. developed an ultra-thin single-electrode triboelectric nanogenerator and integrated it into a wearable self-powered input system (Figure 10a) [107]. Using PDMS as a friction layer, two layers of graphene electrodes were used to generate output signals in x and y directions. The surface of PDMS was treated by plasma to improve the charge density. The overall structure of the device has a negative Poisson ratio auxetic mesh design, which can provide conformal contact on rough surfaces. The TENG tactile sensor can undergo a 13.7% and 8.8% elongation on the x- and y-axes, respectively, while maintaining both mechanical and electrical stability with less than 1% relative resistance and voltage variation, even when subjected to stretching. The touch sensor can clearly detect the touch of a single point and continuous sliding and displayed a character using a real-time trajectory mode. The authors envisaged its potential application in self-powered wearable communication systems. Recently, Cai et al. designed a self-healable (≤2 h), super-stretchable (>760%), and shape-adaptive TENG by doping and modifying PDMS as an e-skin to harvest energy and monitor human motion [108]. In the synthesis of PDMS, cross-linking agent IPDI and chain extender TPAL interact with the raw material to produce hydrogen bonds and imine bonds, which serve as cross-linking points and provide self-healing abilities. Different PDMS friction layers can be obtained by controlling the content of two kinds of bonds. By adding MXene into lightly cross-linked PDMS, a self-healable conductive composite layer was prepared by forming hydrogen bonds between them. The device structure is shown in Figure 10b. This TENG is capable of conforming to the irregular surface of human skin due to its shape adaptability and can retain its original triboelectric performance even after being subjected to repeated damage. The sensor array can be used for real-time monitoring of finger pressure and position. In terms of medical wisdom, Wang et al. designed a self-powered strain sensor based on graphene oxide-polyacrylamide (GO-PAM) hydrogels (Figure 10c) [32]. They mixed graphene oxide into a self-healing hydrogel in order to enhance the conductivity and mechanical properties (toughness and stretchability) of the electrodes. In addition to improving the capacity of charge storage as charge-trapping sites in PAM, GO also causes PAM to produce an obvious fold structure and an enhanced friction area. They placed the strain sensor on an insole, integrated it with a data processing module and PC interface, and utilized a deep learning algorithm to identify and monitor human daily life and pathological gait. The highest recognition accuracy was achieved by the artificial neural network system, with accuracy rates of 99.5% and 98.2% for daily life and pathological gait, respectively (Figure 10c). The researchers envision these smart insoles being utilized to diagnose Parkinson’s disease and hemiplegia, as well to adjust patients’ rehabilitation training based on monitoring data.

Zhang et al. developed a wearable self-powered toroidal triboelectric sensor (STTS) for self-powered human–machine interactions [33]. MXene/Ecoflex nanocomposites and flexible conductive fabric serve as the friction layer with the finger and electrode, respectively (Figure 10d). In order to provide a comfortable contact–separation space between the finger skin and the negatively charged layer, a 3D printed model was used to fabricate pyramidal arrays on the MXene/Ecoflex nanocomposites. Electrical signals are generated when the muscles expand as the finger flexes, increasing the area of contact between the skin and the pyramidal structure. The triboelectric sensor exhibits exceptional properties, including a high peak-to-peak voltage of 19.91 V, an impressive sensitivity of 0.088 VkPa^−1^, and a wide pressure detection range spanning from 0 to 120 kPa. These remarkable attributes enable the generation of high-quality output signals, which facilitate the accurate detection of various finger movements. To achieve excellent flexibility and wearability of the sensor, a glove skeleton was created using 3D printing technology and flexible TPU material, while the fingers were made into a ring structure (Figure 10d). This design allows the single-electrode TENG to be seamlessly attached to the inner surface of the ring, enhancing the user experience. When integrated with a signal transmission processing system, STTS can facilitate human–machine interaction, including control of racing games, switching of home appliances, and playing realistic balance table games.

In addition to monitoring pressure and physical activity, TENGs can also be applied for the detection of respiratory behavior and gases. Wang et al. developed a TENG-based sensor using Ti_3_C_2_T_x_ MXene/amino-functionalized multiwalled carbon nanotubes (MXene/NH_2_-MWCNTs) as the friction layer and electrode (Figure 10e) [109]. The homogeneous distribution of NH_2_-MWCNTs on organic MXene sheets is attributed to the effect of electrostatic adsorption. As the positive friction layer, one end of the nylon film is anchored in the center of the TENG. As air flows, the nylon film moves periodically, driving the TENG to generate electrical signals. As the wind speed increases, the nylon swings up and down more violently, causing more friction charges to be generated and the peak output voltage and current to increase, which can be used to monitor breathing behavior. Support vector machine algorithms were used to identify various breathing patterns, with an average prediction accuracy of 100% (Figure 10e). Additionally, the triboelectric sensor can specifically detect formaldehyde gas concentration by analyzing the change in output voltage, as formaldehyde gas molecules react with oxygen ions and affect the conductivity of NH_2_-MWCNTs. The device exhibits an excellent gas-sensitive response (35% @ 5 ppm), a low detection limit (10 ppb), and a fast response/recovery time (51/57 s) as a self-powered formaldehyde sensor.

### 3.3. 2D Tribotronic Transistors

**Figure 11:**
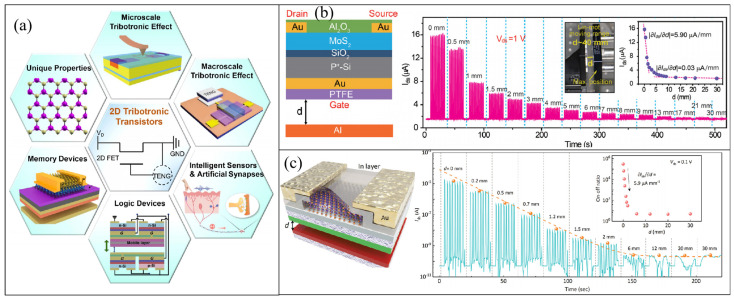
(**a**) TENGs used for 2D tribotronic transistors. Reproduced with permission from ref. [28]. Copyright © 2022 IOP. (**b**) Tactile switch using MoS_2_ as channel material. Reproduced with permission from ref. [113]. Copyright © 2016 Wiley-VCH. (**c**) Tactile switch using InSe as channel material. Reproduced with permission from ref. [114]. Copyright © 2019 Wiley-VCH.

The potential generated by friction in TENGs can also provide an external power source for two-dimensional transistors. Since 2014, when electrostatic potential generated by TENG was used as gate voltage to regulate the electron transport characteristics in a transistor for the first time, the research and application of tribotronic transistor have been continuously deepened [110]. The integration of TENGs in friction transistors involves using one friction layer as a movable layer and the other as an induced voltage, typically placed at the gate of the transistor, to generate potential through friction and regulate the self-powered transistor. By integrating a two-dimensional transistor with a friction nanogenerator into a triboelectronic transistor, the induced friction potential can be used to easily regulate carrier transport characteristics in semiconductor channels. The friction potential generated by low-frequency and intermittent mechanical displacements can be ideal for regulating logic devices [34], photodetectors [111], artificial synapses [112], and other two-dimensional transistor devices (Figure 11a) [28].

Taking basic tactile switches as an example, Wang et al. designed a novel electronic transistor based on a single-motor mode of vertical coupling of a TENG and a MoS_2_ FET [113] for the first time (Figure 11b). They attached a polytetrafluoroethylene (PTFE) film to the back of the FET to act as a friction layer. When PTFE came into contact with the Al electrode or finger skin, it generated a friction voltage, which was used as the gate voltage to regulate the electron concentration in MoS_2_. The contact–separation with the Al electrode is equivalent to generating a negative voltage on the FET gate, thereby reducing the concentration of charge carriers (electrons) in MoS_2_ and the corresponding source-drain current. Meanwhile, with the increase in the separation distance, the negative voltage increases, and the source-drain current continues to decline. When the separation distance increases from 0 to 30 mm, the source-drain current changes by 10 times. In addition, the prepared tribotronic transistor was is used as a smart tactile switch to control the trigger of the transistor through finger contact–separation. The switching ratio can reach 16, which can be used to control the switching state of an LED. Later, Li et al. used InSe as channel material and deposited an In layer on the top of InSe for doping to prepare a tactile switch with higher sensitivity [114] (Figure 11c). As an n-type dopant, the In layer produces a surface charge transfer doping effect and excellent electrical transport characteristics in the conducting channel and protects the device as an encapsulation layer. This also makes the tactile switch more sensitive, and at a low *V*_ds_ of 0.1 V, the switching ratio reaches 10^6^, meeting the requirements of high signal resolution and low power consumption. This can be used to trigger LEDs or demonstrate Morse code.

This kind of self-powered structure can achieve low drive power and low energy loss of the transistor. In addition, using a TENG instead of a traditional external power supply as the grid voltage can better realize the interaction with the external environment and facilitate the preparation of multifunctional and highly integrated device structures [103,113]. Two-dimensional materials possess superior characteristics, such as diverse valence band structures and adjustable thickness, which demonstrate enormous potential in numerous functional tribotronic transistors. Although a TENG integrated in a transistor does not use 2D materials in most of current research, the emergence of high-performance TENGs based on 2D materials integrated in tribotronic transistors can be expected with further research and application.

## 4. Challenges and Prospects

The analysis reported above evidences that TENGs, as an efficient energy conversion method, have attracted the attention of researchers worldwide. Unlike solar energy, wind energy, nuclear energy, and other clean energy sources that are constrained by weather, regional, and safety factors, TENGs can produce energy from various mechanical friction sources anytime and anywhere, with a high energy conversion efficiency. Moreover, unlike PENGs, TENGs do not have any additional structural requirements in terms of materials. The past decade has witnessed the introduction and development of TENGs based on 2D materials. From the initial use of graphene with high conductivity to MXene with high electronegativity, more 2D materials have been used in TENGs, such as Metal organic frameworks (MOFs) and covalent organic frameworks (COFs) [115,116,117]. Besides functioning as electrodes and friction layers, 2D materials are frequently utilized for doping modifications in order to enhance the output performance of TENGs. The research focus has shifted from primarily investigating the performance of TENGs to gradually incorporating them into practical applications, such as energy harvesting, self-powered sensors, and human–machine interaction. Through extensive research efforts, the output performance of TENGs has been significantly improved, and their application field has been expanded. However, in the context of the rapid development of electronic information technology and the IoT, additional challenges and expectations are associated with TENGs.

Although two decades have passed since the first successful stripping of 2D materials, the remarkable magnetic, thermal, piezoelectric, triboelectric, and other properties exhibited by atomic-layer thick materials have not yet been fully explained [118,119]. This is the primary problem limiting the widespread use of such materials. Secondly, the methods available for preparing 2D materials are limited, which poses a serious challenge in the development of large-area, high-quality, and controllable crystal-phase 2D materials at a low cost. Moreover, as electrodes and friction layers in TENGs, 2D materials must fulfill specific requirements in terms of electronegativity and conductivity. Consequently, the development and utilization of new materials similar to MXene (e.g., Ti_3_C_2_T_x_) are also necessary to improve the performance of TENGs. Overall, there remains a need for further research into the properties and production of 2D materials to fully unlock their potential.

Upon review of the preceding summary, it is evident that 2D materials have the potential to serve as modified constituents that can be incorporated into base materials to form composite materials. For instance, they can enhance the dielectric properties of materials, regulate the work function, and facilitate charge capture and interface lubrication. While these benefits have yielded positive results in TENGs, they are macroscopic effects ascertained by various testing instruments, and a robust microscopic mechanism model to explain them is still lacking. Therefore, the establishment of a stable theoretical model for analysis of 2D materials and their interaction with functionalization and base materials is essential to enhance the performance of TENGs.

In terms of applications, the soft, transparent, and ultra-thin properties of 2D materials make them ideal for the preparation of flexible TENGs. However, with the rapid development of IoT technology and the growing demand for wearable devices, there is an increasing need for more flexible, miniaturized, self-powered, and wirelessly transmitted devices. Consequently, TENGs must be designed with clever stacking structures and integrated with more signal processing and transmission units in order to meet specialized requirements such as biological compatibility and environmental stability. Furthermore, the development of hybrid energy harvesters that utilize both triboelectric effects and other effects is necessary to enable signal multimode sensing and to broaden the application field of TENGs based on 2D materials [120].

With regard to the field of TENGs, there is no standardized evaluation system to describe its output performance. Various test factors, such as different test environments, applied loads, contact areas, and device structural parameters, can lead to different results in terms of general test results such as open-circuit voltage, short-circuit current, power density, energy conversion efficiency, etc. [121,122]. Consequently, it is insufficient to form a unified evaluation standard based solely on the number of LED lights and the charging ability of a capacitor, as this is not conducive to the comparative optimization of TENG performance. Moreover, the high impedance of TENGs and the destruction of temperature remain major obstacles that limit their application potential.

## 5. Conclusions

This review provides a comprehensive summary of recent research progress on the TENGs based on 2D materials. From a materials perspective, we reviewed the various roles that 2D materials, including graphene and its derivatives, h-BN, TMDs, and MXene, can play in TENGs, such as serving as electrodes, friction layers, or doping modifications. The applications of TENGs in energy harvesting, self-powered sensing, and human–computer interaction were also discussed. Finally, the challenges and future prospects for the development of TENGs were identified. In conclusion, the successful use of 2D materials in TENGs provides new possibilities to enhance their performance and promote practical applications, thereby advancing the field of self-powered sensing and wearable technology. Further progress towards higher-quality TENGs requires a more substantial theoretical foundation, the development of novel materials, and innovative structural designs.

## Figures and Tables

**Table 1:** Summary pf the performance of TENGs based on 2D materials.

Negative Friction Layer	Positive Friction Layer	Electrode	Output Performance			Refs.
Graphene	PET	Graphene	5 V	0.5 µA cm^−2^	2.5 µW cm^−2^	[46]
AGS@PDMS	Copper foil	Copper foil	117 V	26 µA	480 µW cm^−2^	[47]
GN-PTFE	Al	Cu	96 V	3.66 μA	39 µW cm^−2^	[48]
Silicone	CG	Graphene	9.3 V	-	1.5 μW cm^−2^	[49]
PDMS	CG	CG	83 V	25.78 μA	250 µW cm^−2^	[51]
F-GO/PVDF	water droplet	Cu/ITO	16.5 V	18.1 μA	280 µW cm^−2^	[53]
PI(Kapton)/PI: rGO/PI	Al	Al	190V	-	630 µW cm^−2^	[54]
PVDF/rGO-AgNPs	Al	Al	200 V	-	430 µW cm^−2^	[55]
Al_2_O_3_/BN	Graphene	Graphene	1.2 V	150 nA cm^−2^	-	[68]
BNNSs/BoPET	Paper	Copper	200V	0.48 mA m^−2^	14 µW cm^−2^	[69]
PI/BNNS/PI	Al	Al	65.9 V	4.5 μA	21.4 μW cm^−2^	[70]
PI(Kapton)/MoS_2_:PI/PI	Al	Al	400 V	175 mA m^−2^	2570 µW cm^−2^	[76]
MoS_2_/SiO_2_	PDMS	Al/Si	25 V	1.2 μA	2.25 μW cm^−2^	[79]
LC-WS_2_	PET	ITO	12.2 V	-	13.8 µW cm^−2^	[81]
MXene	PET	ITO	650 V	-	52 µW cm^−2^	[29]
MXene	Skin	MXene	16.4 V	2.67 μA	2.89 µW cm^−2^	[85]
Kapton	Ecoflex	MXene/PVA	230 V	270 nA	-	[87]
MXene/Silicone	hand	Ag fabric	35 V	12.5 µA m^−2^	5.5 µW cm^−2^	[86]
PDMS/MXenes	PDMS/AgNPs	AgNPs	453 V	131 μA	-	[31]
Mg-Al LDH	water droplet	Al	13 V	1.6 μA cm^−2^	-	[93]
ZnAl-LDH-PVDF	PET	ITO	230.6 V	5.6 mA cm^−2^	430 µW cm^−2^	[95]
